# (*S*)-(−)-1-Phenyl­ethanaminium 4-(4,4-di­fluoro-1,3,5,7-tetra­methyl-3a,4a-diaza-4-borata-*s*-indacen-8-yl)benzoate

**DOI:** 10.1107/S1600536811019167

**Published:** 2011-08-06

**Authors:** Lindsay M. Hinkle, Raghu Chitta, Kent R. Mann

**Affiliations:** a Department of Chemistry, University of Minnesota, Minneapolis, MN 55455, USA

## Abstract

The title compound, C_8_H_12_N^+^·C_20_H_18_BF_2_N_2_O_2_^−^, crystallizes with a significant amount of void space [4.0 (5)%] in the unit cell. The structure displays N—H⋯O hydrogen bonding between the components. The plane formed by the benzoic acid moiety of the BODIPY-CO_2_^−^ is twisted by 80.71 (6)° relative to the plane formed by the ring C and N atoms of the tetramethyldipyrrin portion of the molecule.

## Related literature

For the use of crystalline materials that contain emissive transition metal complexes for sensing small mol­ecules, see: McGee & Mann (2007 ▶); Smith & Mann (2009 ▶). The boron dipyrrin family of dyes could be an alternative to these often costly transition metal complexes and can also be easily modified at the *meso* position, see: Erten-Ela *et al.* (2008 ▶); Ulrich *et al.* (2008 ▶). We have found that to sense small mol­ecules effectively, empty channels must be present in the crystal structure to allow the analyte mol­ecules to penetrate the crystalline lattice, see: McGee & Mann (2007 ▶); McGee *et al.* (2007 ▶); Smith & Mann (2009 ▶). For factors that could facilitate inefficient packing, see: Lancaster *et al.* (2006 ▶); Imai *et al.* (2007 ▶, 2008 ▶); Brock *et al.* (1991 ▶); Tominaga *et al.* (2011 ▶). Mol­ecules such as methanol and water have mol­ecular volumes consistent with their possible incorporation in the void cavities, see: Buss *et al.* (1998 ▶) For details of the synthesis, see: Tomasulo *et al.* (2008 ▶). For refinement details, see: Flack (1983 ▶). For a description of the Cambridge Structural Database, see: Allen (2002 ▶). The amount and location of the void space was analyzed with *PLATON*/*VOID* (Spek, 2009) ▶. For Wallach’s rule, see: Herbstein (2005 ▶).
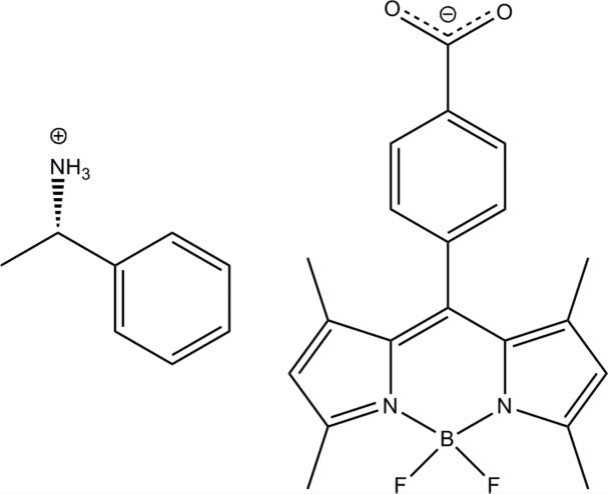


## Experimental

### 

#### Crystal data

C_8_H_12_N^+^·C_20_H_18_BF_2_N_2_O_2_^−^
*M* *_r_* = 489.36
Monoclinic, 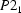

*a* = 12.492 (5) Å
*b* = 6.629 (4) Å
*c* = 16.042 (7) Å
β = 96.74 (3)°
*V* = 1319.2 (11) Å^3^
*Z* = 2
Mo *K*α radiation
μ = 0.09 mm^−1^
*T* = 173 K
0.50 × 0.30 × 0.03 mm

#### Data collection

Siemens SMART Platform CCD diffractometer
Absorption correction: multi-scan (*SADABS*; Sheldrick, 2008*a* ▶) *T*_min_ = 0.958, *T*_max_ = 0.997
11639 measured reflections
4593 independent reflections
3331 reflections with *I* > 2σ(*I*)
*R*_int_ = 0.052

#### Refinement

*R*[*F* ^2^ > 2σ(*F* ^2^)] = 0.049
*wR*(*F* ^2^) = 0.130
*S* = 1.03
4593 reflections
327 parameters
1 restraint
H-atom parameters constrained
Δρ_max_ = 0.23 e Å^−3^
Δρ_min_ = −0.21 e Å^−3^

### 

Data collection: *SMART* (Bruker, 2003 ▶); cell refinement: *SAINT* (Bruker, 2003 ▶); data reduction: *SAINT*; program(s) used to solve structure: *SHELXS97* (Sheldrick, 2008*b* ▶); program(s) used to refine structure: *SHELXL97* (Sheldrick, 2008*b* ▶); molecular graphics: *SHELXTL* (Sheldrick, 2008*b* ▶); software used to prepare material for publication: *SHELXTL*.

## Supplementary Material

Crystal structure: contains datablock(s) I, global. DOI: 10.1107/S1600536811019167/fl2344sup1.cif

Structure factors: contains datablock(s) I. DOI: 10.1107/S1600536811019167/fl2344Isup2.hkl

Additional supplementary materials:  crystallographic information; 3D view; checkCIF report

## Figures and Tables

**Table 1:** Hydrogen-bond geometry (Å, °)

*D*—H⋯*A*	*D*—H	H⋯*A*	*D*⋯*A*	*D*—H⋯*A*
N3—H1*N*3⋯O1^i^	0.87	1.93	2.743 (4)	155
N3—H2*N*3⋯O2	0.87	2.00	2.872 (3)	178
N3—H3*N*3⋯O2^ii^	0.87	1.94	2.801 (3)	169

Symmetry codes: (i) 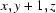; (ii) 
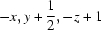
.

**Fig. 1.**
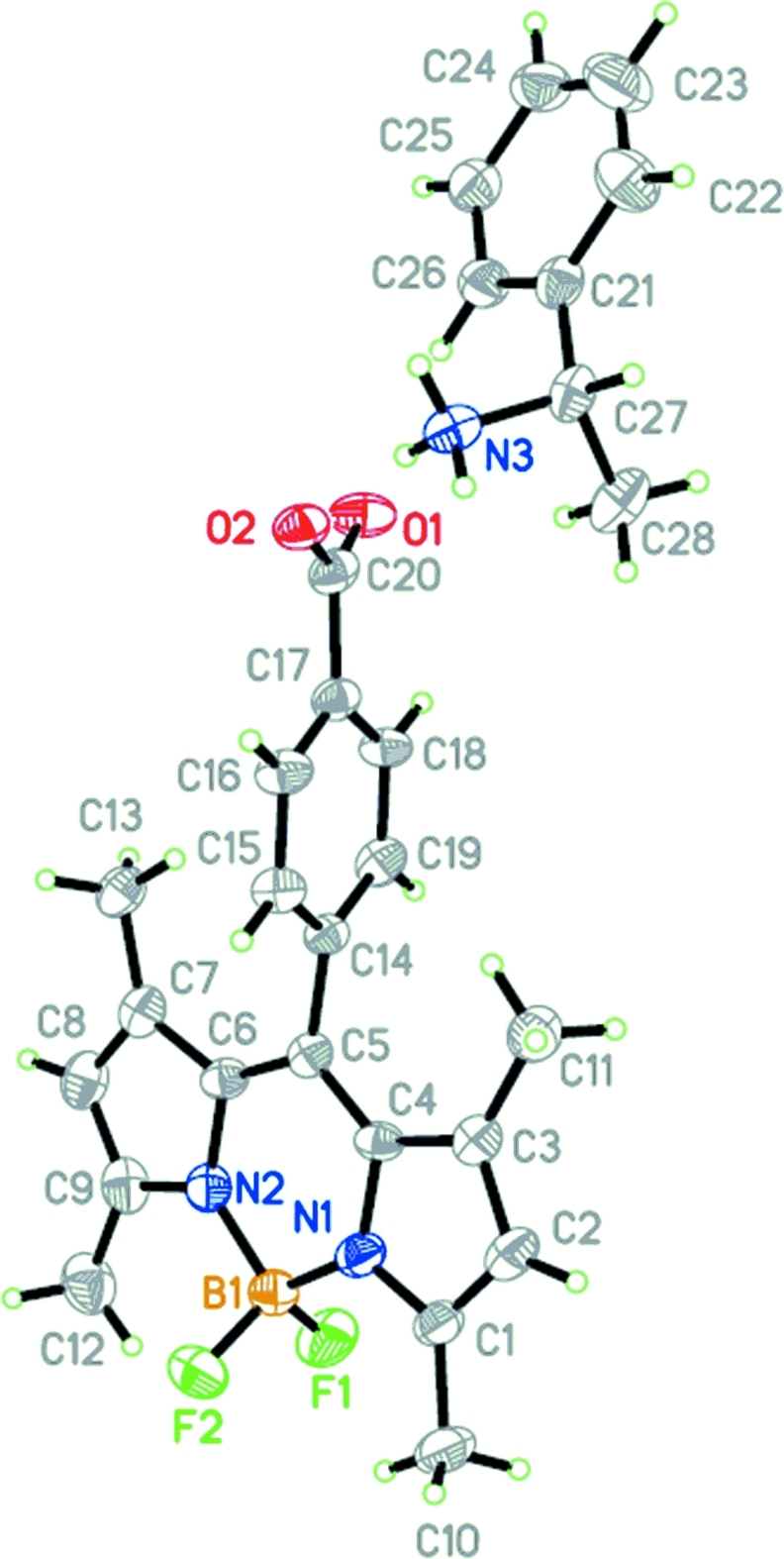
Labeled diagram of the asymmetric unit. Ellipsoids are drawn at 50% probability and methyl hydrogen disorder has been removed for clarity.

**Fig. 2.**
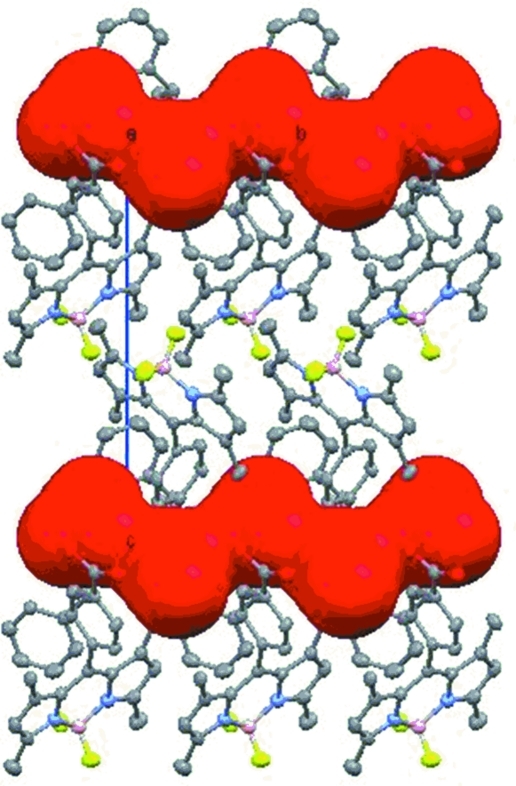
View of the packing down the a axis. Areas of void space are drawn as red spheres. Thermal ellipsoids are drawn at 50% probability and all hydrogen atoms have been removed for clarity.

## supplementary crystallographic information

### Comment

Our group is studying the use of crystalline materials that contain emissive transition metal complexes for sensing small molecules by luminescen quenching (McGee & Mann, 2007; Smith & Mann, 2009). The boron dipyrrin, or BODIPY, family of dyes could be an alternative to these often costly transition metal complexes. BODIPYs possess several desirable qualities including high molar absorptivities and large emission quantum yields. They can also be easily modified at the *meso* position (Erten-Ela *et al.*, 2008; Ulrich *et al.* 2008).

We have found to sense small molecules effectively, empty channels must be present in the crystal structure to allow the analyte molecules to penetrate the crystalline lattice (McGee & Mann, 2007; Smith & Mann, 2009). Void space in the form of channels is often difficult to obtain as nature prefers to pack molecules as efficiently as possible in centrosymmetric space groups. A recent search of the Cambridge Structural Database (CSD, Version 5.3 of November 2009; Allen, 2002) revealed the most commonly observed space groups are centrosymmetric with high packing efficiency.

We hypothesized, based on previous work of Lancaster *et al.*, 2006, Imai *et al.*, 2007, 2008, and Brock *et al.*, 1991, that the co-crystallization of an optically pure chiral amine with the sensing chromophore could facilitate inefficient packing as the molecular chirality requires a non-centrosymmetric space group. To this same end, increasing the number of specific intermolecular interactions (*i.e.* hydrogen bonding, (Tominaga *et al.*, 2011)) could increase the probability of inefficient packing while supporting and strengthening a lattice containing significant amounts of void space. Our initial attempt to apply this hypothesis experimentally was successful and is reported here. X-ray quality crystals of the amine-BODIPY adduct showed both specific intermolecular interactions as well as channels of void space.

The BODIPY-CO_2_H crystallizes with the chiral amine (*S*)-(-)-α-methylbenzylamine in the solid-state by forming an ammonium moiety and a deprotonated carboxylic acid pair linked by hydrogen bonds (Figure 1). Information regarding the hydrogen bonding within this structure can be found in Table 1. It is also possible that this hydrogen is partially occupied on both N3 and O2, but this model did not significantly improve the refinement statistics.

*PLATON*/VOID (Spek, 2009) was used to determine the amount and location of the solvent accessible void space within the structure. Voids of 52 (1) Å^3^ were found in the unit cell which corresponds to 4.0 (5) % of the total unit cell volume. Molecules such as methanol (37 (1) Å^3^) and water (22 (1) Å^3^) have molecular volumes consistent with their possible incorporation in the void cavities (Buss *et al.*, 1998). Figure 2 shows the packing of the structure and the location of the void space as represented by the red spheres. These isolated void channels run parallel to the *b* axis with spokes of void space perpendicular from the main channel. These spokes form around the hydrogen bonding that occurs throughout the structure.

The carbon-oxygen bond lengths of the carboxylate moiety are almost identical (C20—O1 = 1.262 (4) Å and C20—O2 = 1.280 (4) Å). The plane formed by the benzoic acid moiety of the BODIPY-CO_2_^-^ (C14 > C20) is twisted 80.71 (6)° relative to the plane formed by the tetramethyldipyrrin portion of the molecule (N1 N2 C1 > C9).

The hydrogen bonding pattern can be described as two identical parallel tape interactions that are joined by an additional interaction to form a two-dimensional lattice of hydrogen bonding. The first nearly linear tape is described by graph set notation as C^2^_2_(6), the second zigzag tape as C^1^_2_(4), and the ring interaction that results from the hydrogen bond that spans the two tapes as *R*^3^_4_(10).

### Experimental

The 4,4-difluoro-8-(4-carboxyphenyl)-1,3,5,7-tetramethyl-3a,4a-diaza-4-bora-s-indacene (BODIPY-CO_2_H) was synthesized according to Tomasulo *et al.* (2008) and purified on silica gel with hexanes:acetone (60:40 *v*/*v*). BODIPY-CO_2_H (6 mg, 0.016 mmol) was dissolved in CH_2_Cl_2_ (1 ml) and (*S*)-(-)-α-methylbenzylamine (15.3 mg, 0.126 mmol; purchased from Fluka) was added to this solution. After sonication produced a homogenous solution, 1 ml of MeOH was added. X-ray quality crystals were obtained through slow evaporation of this solution. Crystals were washed with hexanes to remove residual amine before data collection.

### Refinement

Attempts to place one hydrogen atom on the carboxylic acid moiety and two on the amine moiety gave poorer refinement statistics. The better model is that shown in Figure 1 as an ion pair.

All aromatic H atoms were placed in ideal positions and refined as riding, with C—H = 0.95 Å and *U*_iso_(H) = 1.2*U*_eq_(C). Methyl H atoms were placed in ideal positions and refined as riding, with C—H = 0.98 Å and *U*_iso_(H) = 1.5*U*_eq_(C). Those methyl H atoms on the BODIPY-CO_2_^-^ molecule were modeled over two postions 60 ° from one another with half occupancy. Hydrogen atoms located on the ammonium moiety were placed in ideal positions and refined as riding, with N—H = 0.87 Å and *U*_iso_(H) = 1.5*U*_eq_(N).

The chirality of the ammonium bearing molecule is known to be *S* at C27 as the *S* isomer of the chiral amine was introduced as a co-crystallant. The Flack *x* parameter (Flack, 1983) based on refinement with 2071 Friedel pairs was 0.4 (10), indicating no conclusions can be drawn regarding the absolute structure and Friedel pairs were merged before final refinement of the structure.

### Crystal data

**Table:**

C_8_H_12_N^+^·C_20_H_18_BF_2_N_2_O_2_^−^	*F*(000) = 516
*M**_r_* = 489.36	*D*_x_ = 1.232 Mg m^−^^3^
Monoclinic, *P*2_1_	Mo *K*α radiation, λ = 0.71073 Å
*a* = 12.492 (5) Å	Cell parameters from 2034 reflections
*b* = 6.629 (4) Å	θ = 2.6–26.8°
*c* = 16.042 (7) Å	µ = 0.09 mm^−^^1^
β = 96.74 (3)°	*T* = 173 K
*V* = 1319.2 (11) Å^3^	Block, orange
*Z* = 2	0.50 × 0.30 × 0.03 mm

### Data collection

**Table:**

Siemens SMART Platform CCD diffractometer	4593 independent reflections
Radiation source: normal-focus sealed tube	3331 reflections with *I* > 2σ(*I*)
graphite	*R*_int_ = 0.052
area detector, ω scans per φ	θ_max_ = 25.0°, θ_min_ = 1.3°
Absorption correction: multi-scan (*SADABS*; Sheldrick, 2008*a*)	*h* = −14→14
*T*_min_ = 0.958, *T*_max_ = 0.997	*k* = −7→7
11639 measured reflections	*l* = −19→19

### Refinement

**Table:**

Refinement on *F*^2^	Primary atom site location: structure-invariant direct methods
Least-squares matrix: full	Secondary atom site location: difference Fourier map
*R*[*F*^2^ > 2σ(*F*^2^)] = 0.049	Hydrogen site location: inferred from neighbouring sites
*wR*(*F*^2^) = 0.130	H-atom parameters constrained
*S* = 1.03	*w* = 1/[σ^2^(*F*_o_^2^) + (0.071*P*)^2^] where *P* = (*F*_o_^2^ + 2*F*_c_^2^)/3
4593 reflections	(Δ/σ)_max_ < 0.001
327 parameters	Δρ_max_ = 0.23 e Å^−^^3^
1 restraint	Δρ_min_ = −0.21 e Å^−^^3^

### Special details

**Table:**

Experimental. Cell errors are from iterative updates since the crystal is believed to have been moving during the data collection.
Geometry. All e.s.d.'s (except the e.s.d. in the dihedral angle between two l.s. planes) are estimated using the full covariance matrix. The cell e.s.d.'s are taken into account individually in the estimation of e.s.d.'s in distances, angles and torsion angles; correlations between e.s.d.'s in cell parameters are only used when they are defined by crystal symmetry. An approximate (isotropic) treatment of cell e.s.d.'s is used for estimating e.s.d.'s involving l.s. planes.
Refinement. Refinement of *F*^2^ against ALL reflections. The weighted *R*-factor *wR* and goodness of fit *S* are based on *F*^2^, conventional *R*-factors *R* are based on *F*, with *F* set to zero for negative *F*^2^. The threshold expression of *F*^2^ > σ(*F*^2^) is used only for calculating *R*-factors(gt) *etc*. and is not relevant to the choice of reflections for refinement. *R*-factors based on *F*^2^ are statistically about twice as large as those based on *F*, and *R*- factors based on ALL data will be even larger.Errors in the CIF check pertaining to the Flack parameter should be ignored. Not only is this structure a light atom structure, but the chirality of the amine is known explicitly from the synthesis of the material to be *S* at C27.This same argument can be used to ignore the other errors regarding the Friedel data. Merging the data with the MERG 4 command did not significantly decrease the number of errors received in the cif report, and in fact made the number of significant errors increase. Consequently, the structure was not refined with the MERG 4 command. The number of Friedel pairs calculated by using the MERG 2 and MERG 4 commands was 2071 which is in very close agreement with the number calculated in this CIF of 2052.

### Fractional atomic coordinates and isotropic or equivalent isotropic displacement parameters (Å^2^)

**Table:**

	*x*	*y*	*z*	*U*_iso_*/*U*_eq_	Occ. (<1)
B1	0.7689 (3)	0.7133 (6)	0.9400 (2)	0.0381 (10)	
O1	0.18036 (17)	0.6656 (3)	0.50847 (14)	0.0419 (6)	
O2	0.12408 (15)	0.9456 (3)	0.56863 (13)	0.0352 (5)	
N1	0.75434 (19)	0.8879 (4)	0.87528 (16)	0.0341 (7)	
N2	0.66680 (19)	0.5761 (4)	0.92351 (15)	0.0332 (6)	
N3	0.08689 (18)	1.2997 (4)	0.46613 (15)	0.0337 (6)	
H1N3	0.1243	1.3954	0.4928	0.051*	
H2N3	0.0955	1.1883	0.4948	0.051*	
H3N3	0.0190	1.3327	0.4598	0.051*	
F1	0.86211 (14)	0.6040 (3)	0.93019 (12)	0.0528 (6)	
F2	0.77597 (15)	0.7887 (3)	1.02279 (11)	0.0529 (5)	
C1	0.8260 (2)	1.0396 (5)	0.8678 (2)	0.0355 (8)	
C2	0.7862 (2)	1.1611 (5)	0.7977 (2)	0.0395 (8)	
H2A	0.8207	1.2778	0.7792	0.047*	
C3	0.6896 (2)	1.0825 (4)	0.76100 (19)	0.0328 (7)	
C4	0.6676 (2)	0.9086 (4)	0.81062 (18)	0.0282 (7)	
C5	0.5803 (2)	0.7722 (4)	0.80429 (18)	0.0277 (7)	
C6	0.5779 (2)	0.6109 (4)	0.86052 (19)	0.0295 (7)	
C7	0.4972 (2)	0.4591 (5)	0.8716 (2)	0.0348 (8)	
C8	0.5405 (3)	0.3394 (5)	0.9380 (2)	0.0414 (9)	
H8A	0.5062	0.2260	0.9595	0.050*	
C9	0.6438 (3)	0.4127 (5)	0.9687 (2)	0.0397 (8)	
C10	0.9285 (3)	1.0655 (6)	0.9256 (2)	0.0513 (10)	
H10A	0.9346	0.9575	0.9676	0.077*	0.50
H10B	0.9278	1.1965	0.9538	0.077*	0.50
H10C	0.9901	1.0593	0.8931	0.077*	0.50
H10D	0.9671	1.1847	0.9087	0.077*	0.50
H10E	0.9739	0.9457	0.9225	0.077*	0.50
H10F	0.9115	1.0829	0.9833	0.077*	0.50
C11	0.6218 (3)	1.1628 (5)	0.6827 (2)	0.0434 (9)	
H11A	0.5569	1.0798	0.6706	0.065*	0.50
H11B	0.6640	1.1574	0.6349	0.065*	0.50
H11C	0.6010	1.3027	0.6922	0.065*	0.50
H11D	0.6577	1.2801	0.6612	0.065*	0.50
H11E	0.5506	1.2025	0.6969	0.065*	0.50
H11F	0.6136	1.0572	0.6396	0.065*	0.50
C12	0.7212 (3)	0.3300 (6)	1.0401 (2)	0.0530 (10)	
H12A	0.7868	0.4123	1.0469	0.080*	0.50
H12B	0.7398	0.1904	1.0277	0.080*	0.50
H12C	0.6871	0.3335	1.0921	0.080*	0.50
H12D	0.6890	0.2119	1.0642	0.080*	0.50
H12E	0.7360	0.4338	1.0835	0.080*	0.50
H12F	0.7887	0.2906	1.0190	0.080*	0.50
C13	0.3869 (2)	0.4326 (5)	0.8233 (2)	0.0378 (8)	
H13A	0.3758	0.5351	0.7791	0.057*	0.50
H13B	0.3318	0.4476	0.8616	0.057*	0.50
H13C	0.3815	0.2980	0.7980	0.057*	0.50
H13D	0.3502	0.3187	0.8467	0.057*	0.50
H13E	0.3943	0.4062	0.7642	0.057*	0.50
H13F	0.3446	0.5558	0.8278	0.057*	0.50
C14	0.4866 (2)	0.7945 (5)	0.73731 (18)	0.0269 (7)	
C15	0.4026 (2)	0.9297 (5)	0.7490 (2)	0.0326 (7)	
H15A	0.4089	1.0165	0.7964	0.039*	
C16	0.3101 (2)	0.9367 (5)	0.69111 (19)	0.0340 (8)	
H16A	0.2543	1.0291	0.6998	0.041*	
C17	0.2975 (2)	0.8110 (5)	0.62063 (19)	0.0298 (7)	
C18	0.3830 (2)	0.6827 (4)	0.60732 (19)	0.0307 (7)	
H18A	0.3778	0.5996	0.5588	0.037*	
C19	0.4763 (2)	0.6762 (5)	0.6652 (2)	0.0341 (8)	
H19A	0.5337	0.5891	0.6549	0.041*	
C20	0.1934 (2)	0.8065 (5)	0.56131 (19)	0.0329 (7)	
C21	0.0487 (2)	1.1316 (4)	0.3262 (2)	0.0332 (8)	
C22	−0.0024 (3)	1.1990 (6)	0.2490 (2)	0.0523 (10)	
H22A	0.0091	1.3334	0.2314	0.063*	
C23	−0.0698 (3)	1.0721 (7)	0.1975 (2)	0.0626 (11)	
H23A	−0.1026	1.1198	0.1447	0.075*	
C24	−0.0894 (3)	0.8777 (6)	0.2223 (2)	0.0460 (9)	
H24A	−0.1359	0.7919	0.1872	0.055*	
C25	−0.0400 (2)	0.8084 (6)	0.2998 (2)	0.0419 (8)	
H25A	−0.0546	0.6758	0.3179	0.050*	
C26	0.0303 (2)	0.9322 (5)	0.3508 (2)	0.0360 (8)	
H26A	0.0657	0.8818	0.4022	0.043*	
C27	0.1249 (2)	1.2695 (5)	0.38100 (19)	0.0355 (8)	
H27A	0.1254	1.4040	0.3528	0.043*	
C28	0.2423 (3)	1.1901 (6)	0.3944 (2)	0.0503 (10)	
H28A	0.2703	1.1790	0.3400	0.075*	
H28B	0.2436	1.0572	0.4212	0.075*	
H28C	0.2872	1.2840	0.4305	0.075*	

### Atomic displacement parameters (Å^2^)

**Table:**

	*U*^11^	*U*^22^	*U*^33^	*U*^12^	*U*^13^	*U*^23^
B1	0.031 (2)	0.047 (2)	0.035 (2)	0.0018 (18)	−0.0001 (18)	0.0033 (19)
O1	0.0409 (13)	0.0309 (12)	0.0498 (15)	−0.0021 (10)	−0.0126 (11)	−0.0074 (11)
O2	0.0266 (11)	0.0357 (12)	0.0418 (13)	0.0041 (10)	−0.0020 (10)	0.0034 (11)
N1	0.0251 (14)	0.0388 (16)	0.0381 (16)	−0.0008 (12)	0.0020 (13)	−0.0019 (12)
N2	0.0304 (15)	0.0352 (15)	0.0343 (15)	0.0052 (12)	0.0050 (13)	0.0051 (13)
N3	0.0267 (13)	0.0294 (14)	0.0438 (15)	−0.0009 (12)	−0.0005 (12)	−0.0014 (13)
F1	0.0297 (10)	0.0592 (13)	0.0687 (13)	0.0124 (9)	0.0027 (10)	0.0105 (11)
F2	0.0584 (13)	0.0623 (13)	0.0363 (11)	−0.0085 (11)	−0.0020 (10)	−0.0023 (10)
C1	0.0268 (17)	0.0419 (19)	0.0379 (19)	−0.0046 (16)	0.0047 (15)	−0.0066 (17)
C2	0.0328 (19)	0.0349 (18)	0.052 (2)	−0.0082 (16)	0.0087 (17)	−0.0048 (18)
C3	0.0311 (17)	0.0290 (17)	0.0389 (18)	0.0012 (14)	0.0064 (15)	−0.0026 (15)
C4	0.0239 (16)	0.0301 (17)	0.0291 (17)	0.0049 (14)	−0.0028 (14)	−0.0018 (14)
C5	0.0251 (16)	0.0257 (16)	0.0330 (17)	0.0046 (13)	0.0056 (14)	−0.0031 (14)
C6	0.0238 (16)	0.0296 (17)	0.0343 (17)	0.0046 (13)	0.0005 (14)	−0.0018 (14)
C7	0.0338 (18)	0.0289 (17)	0.043 (2)	0.0017 (15)	0.0103 (16)	−0.0016 (15)
C8	0.045 (2)	0.0311 (18)	0.050 (2)	−0.0004 (16)	0.0142 (18)	0.0072 (17)
C9	0.042 (2)	0.0372 (19)	0.041 (2)	0.0118 (16)	0.0102 (17)	0.0093 (17)
C10	0.0333 (19)	0.063 (2)	0.056 (2)	−0.0136 (19)	0.0016 (18)	−0.013 (2)
C11	0.0370 (19)	0.041 (2)	0.051 (2)	0.0012 (16)	0.0022 (17)	0.0151 (17)
C12	0.057 (2)	0.056 (2)	0.046 (2)	0.012 (2)	0.0080 (19)	0.0130 (19)
C13	0.0349 (18)	0.0301 (17)	0.049 (2)	−0.0040 (15)	0.0084 (16)	0.0002 (16)
C14	0.0242 (16)	0.0241 (15)	0.0316 (17)	0.0002 (13)	−0.0002 (14)	0.0050 (14)
C15	0.0310 (17)	0.0293 (16)	0.0366 (19)	0.0034 (14)	−0.0001 (15)	−0.0054 (15)
C16	0.0270 (17)	0.0322 (17)	0.0419 (19)	0.0077 (14)	−0.0001 (15)	−0.0080 (16)
C17	0.0272 (16)	0.0257 (16)	0.0355 (17)	−0.0024 (14)	−0.0003 (14)	0.0030 (15)
C18	0.0276 (17)	0.0291 (16)	0.0338 (18)	0.0026 (14)	−0.0034 (15)	−0.0069 (14)
C19	0.0280 (17)	0.0302 (17)	0.045 (2)	0.0046 (14)	0.0066 (16)	−0.0038 (16)
C20	0.0283 (17)	0.0266 (17)	0.043 (2)	−0.0045 (15)	0.0014 (15)	0.0068 (17)
C21	0.0281 (17)	0.0317 (18)	0.041 (2)	0.0054 (13)	0.0091 (15)	0.0045 (15)
C22	0.063 (3)	0.044 (2)	0.048 (2)	0.009 (2)	0.001 (2)	0.0098 (19)
C23	0.064 (3)	0.077 (3)	0.044 (2)	0.011 (2)	−0.006 (2)	0.012 (2)
C24	0.039 (2)	0.057 (2)	0.040 (2)	−0.0001 (17)	−0.0010 (17)	−0.0107 (18)
C25	0.0387 (19)	0.045 (2)	0.042 (2)	−0.0037 (17)	0.0055 (17)	−0.0009 (18)
C26	0.0354 (18)	0.0354 (18)	0.0366 (19)	−0.0029 (16)	0.0019 (16)	0.0021 (16)
C27	0.0332 (17)	0.0316 (17)	0.0436 (19)	−0.0035 (14)	0.0124 (16)	0.0035 (15)
C28	0.0337 (19)	0.049 (2)	0.071 (3)	−0.0027 (17)	0.0181 (19)	−0.006 (2)

### Geometric parameters (Å, °)

**Table:**

B1—F1	1.396 (4)	C12—H12A	0.9800
B1—F2	1.413 (4)	C12—H12B	0.9800
B1—N1	1.551 (5)	C12—H12C	0.9800
B1—N2	1.563 (5)	C12—H12D	0.9800
O1—C20	1.259 (4)	C12—H12E	0.9800
O2—C20	1.279 (4)	C12—H12F	0.9800
N1—C1	1.361 (4)	C13—H13A	0.9800
N1—C4	1.416 (4)	C13—H13B	0.9800
N2—C9	1.353 (4)	C13—H13C	0.9800
N2—C6	1.430 (4)	C13—H13D	0.9800
N3—C27	1.511 (4)	C13—H13E	0.9800
N3—H1N3	0.8701	C13—H13F	0.9800
N3—H2N3	0.8701	C14—C19	1.391 (4)
N3—H3N3	0.8701	C14—C15	1.409 (4)
C1—C2	1.424 (5)	C15—C16	1.395 (4)
C1—C10	1.500 (5)	C15—H15A	0.9500
C2—C3	1.381 (4)	C16—C17	1.399 (4)
C2—H2A	0.9500	C16—H16A	0.9500
C3—C4	1.445 (4)	C17—C18	1.401 (4)
C3—C11	1.526 (4)	C17—C20	1.520 (4)
C4—C5	1.410 (4)	C18—C19	1.403 (4)
C5—C6	1.402 (4)	C18—H18A	0.9500
C5—C14	1.501 (4)	C19—H19A	0.9500
C6—C7	1.450 (4)	C21—C22	1.398 (5)
C7—C8	1.386 (4)	C21—C26	1.406 (4)
C7—C13	1.510 (4)	C21—C27	1.523 (4)
C8—C9	1.412 (5)	C22—C23	1.391 (5)
C8—H8A	0.9500	C22—H22A	0.9500
C9—C12	1.513 (5)	C23—C24	1.379 (6)
C10—H10A	0.9800	C23—H23A	0.9500
C10—H10B	0.9800	C24—C25	1.398 (4)
C10—H10C	0.9800	C24—H24A	0.9500
C10—H10D	0.9800	C25—C26	1.395 (5)
C10—H10E	0.9800	C25—H25A	0.9500
C10—H10F	0.9800	C26—H26A	0.9500
C11—H11A	0.9800	C27—C28	1.548 (4)
C11—H11B	0.9800	C27—H27A	1.0000
C11—H11C	0.9800	C28—H28A	0.9800
C11—H11D	0.9800	C28—H28B	0.9800
C11—H11E	0.9800	C28—H28C	0.9800
C11—H11F	0.9800		
			
F1—B1—F2	109.2 (3)	C9—C12—H12C	109.5
F1—B1—N1	110.2 (3)	H12A—C12—H12C	109.5
F2—B1—N1	110.8 (3)	H12B—C12—H12C	109.5
F1—B1—N2	110.7 (3)	C9—C12—H12D	109.5
F2—B1—N2	109.0 (3)	H12A—C12—H12D	141.1
N1—B1—N2	107.0 (3)	H12B—C12—H12D	56.3
C1—N1—C4	108.6 (3)	H12C—C12—H12D	56.3
C1—N1—B1	125.7 (3)	C9—C12—H12E	109.5
C4—N1—B1	125.6 (3)	H12A—C12—H12E	56.3
C9—N2—C6	108.2 (3)	H12B—C12—H12E	141.1
C9—N2—B1	126.5 (3)	H12C—C12—H12E	56.3
C6—N2—B1	125.2 (3)	H12D—C12—H12E	109.5
C27—N3—H1N3	109.5	C9—C12—H12F	109.5
C27—N3—H2N3	109.5	H12A—C12—H12F	56.3
H1N3—N3—H2N3	109.5	H12B—C12—H12F	56.3
C27—N3—H3N3	109.5	H12C—C12—H12F	141.1
H1N3—N3—H3N3	109.5	H12D—C12—H12F	109.5
H2N3—N3—H3N3	109.5	H12E—C12—H12F	109.5
N1—C1—C2	108.4 (3)	C7—C13—H13A	109.5
N1—C1—C10	123.6 (3)	C7—C13—H13B	109.5
C2—C1—C10	128.1 (3)	H13A—C13—H13B	109.5
C3—C2—C1	109.3 (3)	C7—C13—H13C	109.5
C3—C2—H2A	125.4	H13A—C13—H13C	109.5
C1—C2—H2A	125.4	H13B—C13—H13C	109.5
C2—C3—C4	106.2 (3)	C7—C13—H13D	109.5
C2—C3—C11	126.3 (3)	H13A—C13—H13D	141.1
C4—C3—C11	127.4 (3)	H13B—C13—H13D	56.3
C5—C4—N1	120.6 (3)	H13C—C13—H13D	56.3
C5—C4—C3	131.9 (3)	C7—C13—H13E	109.5
N1—C4—C3	107.6 (3)	H13A—C13—H13E	56.3
C6—C5—C4	121.2 (3)	H13B—C13—H13E	141.1
C6—C5—C14	117.5 (3)	H13C—C13—H13E	56.3
C4—C5—C14	121.3 (3)	H13D—C13—H13E	109.5
C5—C6—N2	120.3 (3)	C7—C13—H13F	109.5
C5—C6—C7	132.6 (3)	H13A—C13—H13F	56.3
N2—C6—C7	107.1 (3)	H13B—C13—H13F	56.3
C8—C7—C6	106.1 (3)	H13C—C13—H13F	141.1
C8—C7—C13	125.3 (3)	H13D—C13—H13F	109.5
C6—C7—C13	128.6 (3)	H13E—C13—H13F	109.5
C7—C8—C9	109.2 (3)	C19—C14—C15	118.1 (3)
C7—C8—H8A	125.4	C19—C14—C5	121.9 (3)
C9—C8—H8A	125.4	C15—C14—C5	119.9 (3)
N2—C9—C8	109.4 (3)	C16—C15—C14	120.3 (3)
N2—C9—C12	122.7 (3)	C16—C15—H15A	119.9
C8—C9—C12	128.0 (3)	C14—C15—H15A	119.9
C1—C10—H10A	109.5	C15—C16—C17	121.6 (3)
C1—C10—H10B	109.5	C15—C16—H16A	119.2
H10A—C10—H10B	109.5	C17—C16—H16A	119.2
C1—C10—H10C	109.5	C16—C17—C18	118.0 (3)
H10A—C10—H10C	109.5	C16—C17—C20	121.6 (3)
H10B—C10—H10C	109.5	C18—C17—C20	120.3 (3)
C1—C10—H10D	109.5	C17—C18—C19	120.5 (3)
H10A—C10—H10D	141.1	C17—C18—H18A	119.8
H10B—C10—H10D	56.3	C19—C18—H18A	119.8
H10C—C10—H10D	56.3	C14—C19—C18	121.4 (3)
C1—C10—H10E	109.5	C14—C19—H19A	119.3
H10A—C10—H10E	56.3	C18—C19—H19A	119.3
H10B—C10—H10E	141.1	O1—C20—O2	124.1 (3)
H10C—C10—H10E	56.3	O1—C20—C17	118.0 (3)
H10D—C10—H10E	109.5	O2—C20—C17	117.9 (3)
C1—C10—H10F	109.5	C22—C21—C26	118.5 (3)
H10A—C10—H10F	56.3	C22—C21—C27	120.6 (3)
H10B—C10—H10F	56.3	C26—C21—C27	120.9 (3)
H10C—C10—H10F	141.1	C23—C22—C21	120.9 (4)
H10D—C10—H10F	109.5	C23—C22—H22A	119.5
H10E—C10—H10F	109.5	C21—C22—H22A	119.5
C3—C11—H11A	109.5	C24—C23—C22	120.6 (4)
C3—C11—H11B	109.5	C24—C23—H23A	119.7
H11A—C11—H11B	109.5	C22—C23—H23A	119.7
C3—C11—H11C	109.5	C23—C24—C25	119.3 (3)
H11A—C11—H11C	109.5	C23—C24—H24A	120.4
H11B—C11—H11C	109.5	C25—C24—H24A	120.4
C3—C11—H11D	109.5	C26—C25—C24	120.6 (3)
H11A—C11—H11D	141.1	C26—C25—H25A	119.7
H11B—C11—H11D	56.3	C24—C25—H25A	119.7
H11C—C11—H11D	56.3	C25—C26—C21	120.0 (3)
C3—C11—H11E	109.5	C25—C26—H26A	120.0
H11A—C11—H11E	56.3	C21—C26—H26A	120.0
H11B—C11—H11E	141.1	N3—C27—C21	111.1 (2)
H11C—C11—H11E	56.3	N3—C27—C28	108.2 (3)
H11D—C11—H11E	109.5	C21—C27—C28	113.2 (3)
C3—C11—H11F	109.5	N3—C27—H27A	108.1
H11A—C11—H11F	56.3	C21—C27—H27A	108.1
H11B—C11—H11F	56.3	C28—C27—H27A	108.1
H11C—C11—H11F	141.1	C27—C28—H28A	109.5
H11D—C11—H11F	109.5	C27—C28—H28B	109.5
H11E—C11—H11F	109.5	H28A—C28—H28B	109.5
C9—C12—H12A	109.5	C27—C28—H28C	109.5
C9—C12—H12B	109.5	H28A—C28—H28C	109.5
H12A—C12—H12B	109.5	H28B—C28—H28C	109.5
			
F1—B1—N1—C1	−58.8 (4)	N2—C6—C7—C8	−1.2 (3)
F2—B1—N1—C1	62.1 (4)	C5—C6—C7—C13	−0.6 (5)
N2—B1—N1—C1	−179.3 (3)	N2—C6—C7—C13	177.9 (3)
F1—B1—N1—C4	116.8 (3)	C6—C7—C8—C9	0.6 (3)
F2—B1—N1—C4	−122.3 (3)	C13—C7—C8—C9	−178.5 (3)
N2—B1—N1—C4	−3.7 (4)	C6—N2—C9—C8	−0.9 (3)
F1—B1—N2—C9	63.6 (4)	B1—N2—C9—C8	175.4 (3)
F2—B1—N2—C9	−56.4 (4)	C6—N2—C9—C12	179.0 (3)
N1—B1—N2—C9	−176.2 (3)	B1—N2—C9—C12	−4.7 (5)
F1—B1—N2—C6	−120.6 (3)	C7—C8—C9—N2	0.2 (4)
F2—B1—N2—C6	119.3 (3)	C7—C8—C9—C12	−179.7 (3)
N1—B1—N2—C6	−0.5 (4)	C6—C5—C14—C19	−78.9 (3)
C4—N1—C1—C2	0.0 (3)	C4—C5—C14—C19	101.3 (4)
B1—N1—C1—C2	176.2 (3)	C6—C5—C14—C15	97.2 (3)
C4—N1—C1—C10	−179.8 (3)	C4—C5—C14—C15	−82.6 (3)
B1—N1—C1—C10	−3.6 (5)	C19—C14—C15—C16	2.7 (4)
N1—C1—C2—C3	−0.7 (3)	C5—C14—C15—C16	−173.5 (3)
C10—C1—C2—C3	179.1 (3)	C14—C15—C16—C17	0.2 (5)
C1—C2—C3—C4	1.0 (3)	C15—C16—C17—C18	−2.8 (4)
C1—C2—C3—C11	−178.0 (3)	C15—C16—C17—C20	175.1 (3)
C1—N1—C4—C5	−179.0 (2)	C16—C17—C18—C19	2.5 (4)
B1—N1—C4—C5	4.8 (4)	C20—C17—C18—C19	−175.5 (3)
C1—N1—C4—C3	0.6 (3)	C15—C14—C19—C18	−3.1 (4)
B1—N1—C4—C3	−175.6 (3)	C5—C14—C19—C18	173.1 (3)
C2—C3—C4—C5	178.6 (3)	C17—C18—C19—C14	0.5 (4)
C11—C3—C4—C5	−2.4 (5)	C16—C17—C20—O1	−167.4 (3)
C2—C3—C4—N1	−1.0 (3)	C18—C17—C20—O1	10.5 (4)
C11—C3—C4—N1	177.9 (3)	C16—C17—C20—O2	12.2 (4)
N1—C4—C5—C6	−1.2 (4)	C18—C17—C20—O2	−170.0 (3)
C3—C4—C5—C6	179.2 (3)	C26—C21—C22—C23	−0.1 (5)
N1—C4—C5—C14	178.7 (2)	C27—C21—C22—C23	178.2 (3)
C3—C4—C5—C14	−0.9 (5)	C21—C22—C23—C24	1.3 (6)
C4—C5—C6—N2	−2.8 (4)	C22—C23—C24—C25	−0.5 (5)
C14—C5—C6—N2	177.3 (2)	C23—C24—C25—C26	−1.5 (5)
C4—C5—C6—C7	175.5 (3)	C24—C25—C26—C21	2.7 (5)
C14—C5—C6—C7	−4.3 (4)	C22—C21—C26—C25	−1.9 (4)
C9—N2—C6—C5	−180.0 (2)	C27—C21—C26—C25	179.9 (3)
B1—N2—C6—C5	3.6 (4)	C22—C21—C27—N3	121.2 (3)
C9—N2—C6—C7	1.3 (3)	C26—C21—C27—N3	−60.6 (4)
B1—N2—C6—C7	−175.1 (3)	C22—C21—C27—C28	−116.8 (3)
C5—C6—C7—C8	−179.7 (3)	C26—C21—C27—C28	61.4 (4)

### Hydrogen-bond geometry (Å, °)

**Table:**

*D*—H···*A*	*D*—H	H···*A*	*D*···*A*	*D*—H···*A*
N3—H1N3···O1^i^	0.87	1.93	2.743 (4)	155
N3—H2N3···O2	0.87	2.00	2.872 (3)	178
N3—H3N3···O2^ii^	0.87	1.94	2.801 (3)	169

Symmetry codes: (i) *x*, *y*+1, *z*; (ii) −*x*, *y*+1/2, −*z*+1.
